# Nanomechanical Phenotype of Melanoma Cells Depends Solely on the Amount of Endogenous Pigment in the Cells

**DOI:** 10.3390/ijms19020607

**Published:** 2018-02-18

**Authors:** Michal Sarna, Andrzej Zadlo, Barbara Czuba-Pelech, Krystyna Urbanska

**Affiliations:** Department of Biophysics, Faculty of Biochemistry, Biophysics and Biotechnology, Jagiellonian University, Gronostajowa 7, 30-387 Krakow, Poland; andrzej.zadlo@uj.edu.pl (A.Z.); barbara.czuba-pelech@uj.edu.pl (B.C.-P.); krystyna.urbanska@uj.edu.pl (K.U.)

**Keywords:** cancer cells, cell elasticity, nanomechanical phenotype, metastatic behavior, melanoma, melanin pigment

## Abstract

Cancer cells have unique nanomechanical properties, i.e., they behave as if they were elastic. This property of cancer cells is believed to be one of the main reasons for their facilitated ability to spread and metastasize. Thus, the so-called nanomechanical phenotype of cancer cells is viewed as an important indicator of the cells’ metastatic behavior. One of the most highly metastatic cancer cells are melanoma cells, which have a very unusual property: they can synthesize the pigment melanin in large amounts, becoming heavily pigmented. So far, the role of melanin in melanoma remains unclear, particularly the impact of the pigment on metastatic behavior of melanoma cells. Importantly, until recently the potential mechanical role of melanin in melanoma metastasis was completely ignored. In this work, we examined melanoma cells isolated from hamster tumors containing endogenous melanin pigment. Applying an array of advanced microscopy and spectroscopy techniques, we determined that melanin is the dominating factor responsible for the mechanical properties of melanoma cells. Our results indicate that the nanomechanical phenotype of melanoma cells may be a reliable marker of the cells’ metastatic behavior and point to the important mechanical role of melanin in the process of metastasis of melanoma.

## 1. Introduction

Primary tumors represent a set of heterogeneous cell subsets with distinct properties [1]. Although most cancer cells originate from a single, transformed cell, spontaneous mutations during cancer progression result in differences between individual cells [2]. As a result, only a small fraction of cells acquires the necessary properties to undergo invasion [3]. For years, the search was undertaken to identify cellular markers that would distinguish metastatic from non-metastatic cells. Such markers would allow precise screening of the cells, which ultimately could lead to better diagnosis. Recently, elasticity of cancer cells has shown considerable promise in this regard. This is justified by high specificity of elasticity measurements and unique character of such a cellular marker. Thus, many studies have demonstrated that cancer cells with lower values of the Young’s modulus—the measure of elasticity—also exhibited higher invasive potential [4,5,6]. Based on such observations the so called “nanomechanical phenotype” of cancer cells is viewed as an important indicator of the cells’ metastatic behavior and has even been proposed as a potential diagnostic marker of cancer [7]. 

One of the most highly metastatic cancer cells are melanoma cells, which have a very unusual property: they can synthesize the pigment melanin in large amounts, becoming heavily pigmented [8]. It should be emphasized that melanin synthesis is a heterogeneous process, which leads to different levels of cell pigmentation in melanoma tumors [9]. For years, the role of melanin in melanoma, particularly the impact of melanin pigment on melanoma cells’ metastatic behavior, was under extensive scrutiny with the outcomes so far being unsatisfactory [10]. Moreover, until recently the mechanical effect of melanin presence on the metastatic abilities of melanoma cells was not taken into consideration at all. Importantly, melanin in pigmented cells, including melanoma cells, is in the form of granule-like organelles called melanosomes [11]. These organelles were recently found to have unusual mechanical properties, being very stiff and hard to deform [12,13]. Although the mechanical effect of melanin presence on the elasticity of pigmented melanoma cells in vitro have already been published (e.g., [14,15]), in these studies, melanin pigmentation had to be induced by chemical stimuli. Importantly, no studies have yet reported the mechanical effect of melanin in melanoma cells containing endogenous pigment.

In this work, we examined Bomirski hamster melanoma (BHM) cells isolated from hamster tumors containing endogenous melanin pigment. Melanotic (BHM Ma) and amelanotic (BHM Ab) cells were analyzed. Applying atomic force microscopy and spectroscopy techniques together with advanced biophysical methods, we showed that the nanomechanical phenotype of melanoma cells depends solely on the amount of endogenous pigment in the cells. 

## 2. Results

### 2.1. Melanin Determination in Primary Melanoma Cells 

**Figure 1:**
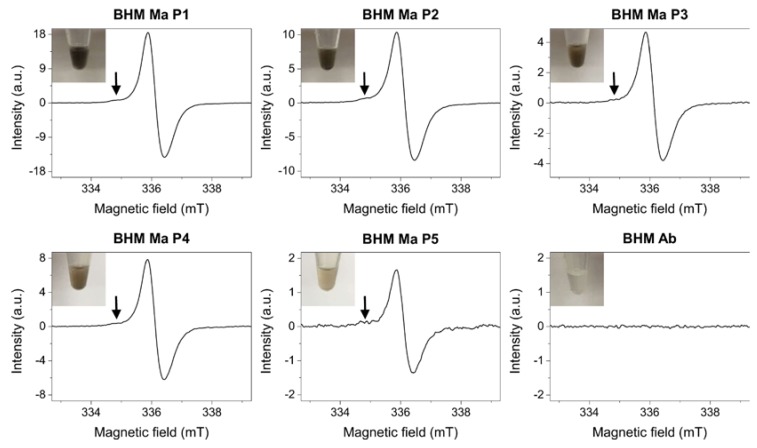
Electron paramagnetic resonance (EPR) spectra of cells examined in this work. Arrows indicate the lower field component attributed to the pheomelanin pigment. Signal intensities are extended to maximum to better show the contribution of pheomelanin component to the EPR melanin spectra. Although the pheomelanin signal seems almost negligible it is worth noting that the detection sensitivity of EPR for pheomelanin determination is nearly an order of magnitude lower than that of eumelanin making the overall contribution of this pigment substantial. Insets show images of cell pallets taken before the analysis.

To determine the amount of endogenous pigment in the cells, electron paramagnetic resonance (EPR) spectroscopy [16] was employed. Figure 1 shows results obtained from EPR analysis. 

As evident from the data, magnetic parameters of the EPR signals obtained for BHM Ma cells were typical for those of eumelanin pigment [17]. Moreover, in these spectra, a lower field component attributed to the pheomelanin pigment [18] could also be observed. On the other hand, no EPR signal of melanin free radicals was detected for BHM Ab cells, which confirms that these cells were amelanotic. Noticeably, signal intensities for BHM Ma cells decreased with each passage. This indicates that the cells, which did not synthesize melanin in vitro, became less pigmented as a result of consecutive divisions. Numerical values of the amount of melanin per cell determined by EPR spectroscopy for each cell sample are shown in Table 1.

### 2.2. Proliferation Abilities of BHM Cells

**Figure 2:**
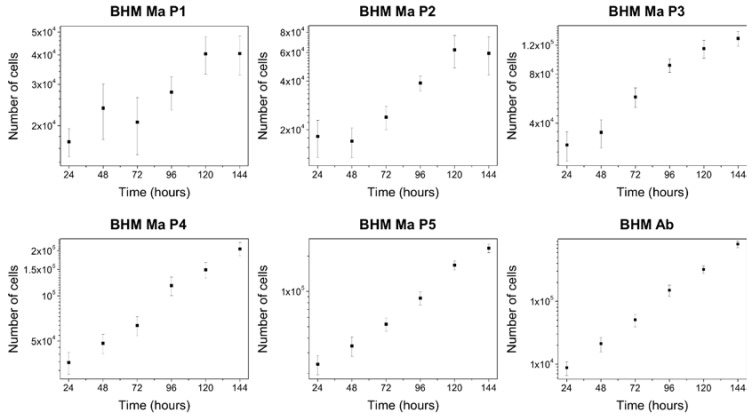
Growth curves of BHM cells presented in a logarithmic scale. Note that BHM Ma cells from first passage have a very uneven distribution with high dispersion of the data, which over consecutive passages becomes more linear and ordered. On the other hand, BHM Ab cells show a very stable growth within the time frame of the experiment. Error bars represent s.d.

To determine the doubling time of the cells, proliferation assay was performed. Figure 2 shows growth curves of the cells examined in this work. 

As evident from the growth curves, pigmented BHM Ma cells from first passage had a very uneven distribution, which over subsequent passages stabilized and resembled that of non-pigmented BHM Ab cells. Numerical values of the doubling times determined for the cells are shown in Table 1. These results clearly indicate that BHM Ma cells had a much slower growth rate than BHM Ab cells. To determine to what extent the observed effect was connected to melanin presence in the cells, relationship graph between melanin content and doubling time of the cells was made. Supplementary Materials Figure S1 shows no direct correlation between melanin presence and proliferation abilities of BHM Ma cells. This suggests that the observed effect was most likely due to the fact that freshly isolated BHM Ma cells needed more time to adapt to in vitro conditions, which is a common effect for primary cell cultures [19]. 

### 2.3. Organization of Cell Cytoskeleton in BHM Cells

**Figure 3:**
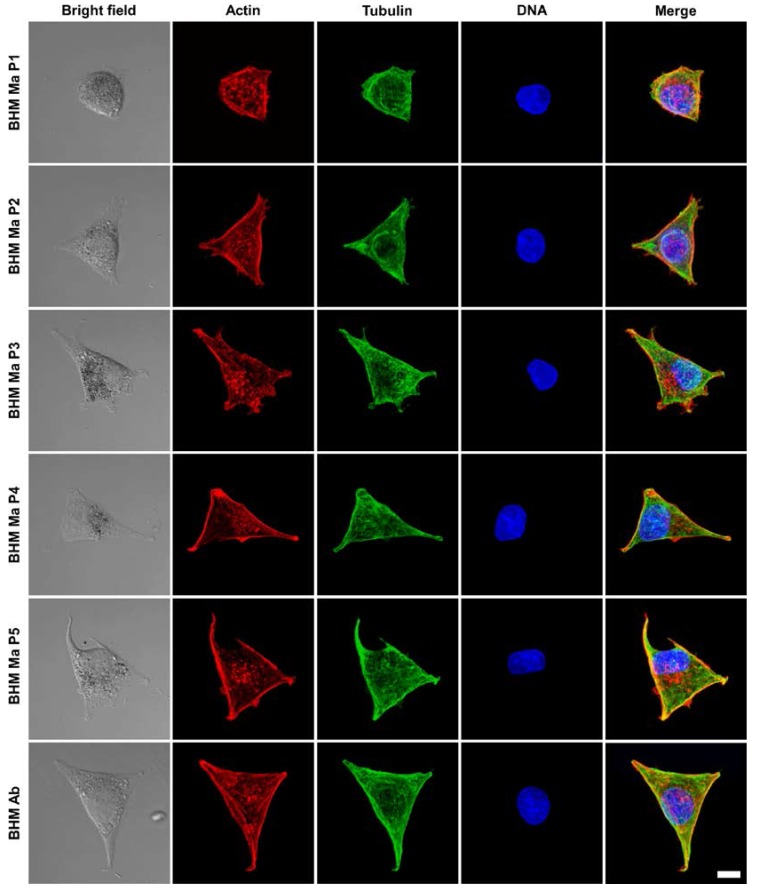
Representative confocal microscopy images of the examined cells. Scatter laser light images (first column from the left) showing the morphology of the cells followed by fluorescence images (remaining columns) of the cells’ cytoskeleton shown in the maximum intensity projection mode. Scale bar for all images represents 10 µm.

To examine the organization of cell cytoskeleton, laser scanning confocal microscopy (LSCM) analysis was performed. Figure 3 shows representative images of BHM cells obtained with LSCM. 

As evident from the images, BHM Ma cells from first passage had a more rounded morphology than cells from later passages. Detailed confocal microscopy analysis revealed that cells from early passages were much higher and less spread than cells from later passages (Supplementary Materials Figure S2). Moreover, confocal microscopy images, taken at different focusing levels of the cells, showed that the actin cytoskeleton of BHM Ma cells from early passages was less developed than that of cells from later passages and of BHM Ab cells (Supplementary Materials Figure S3). In BHM Ma cells, actin filaments were more prominent in the case of cells from later passages and resembled those of BHM Ab cells. This indicates that BHM Ma cells from early passages were less attached to the substrate, hence their actin cytoskeleton was less developed, and this is why they were less spread than cells from later passages. On the other hand, microtubule organization was very similar between the cells, i.e., microtubules extended uniformly throughout the cell body in all examined cells. 

### 2.4. Nanomechanical Properties of BHM Cells

**Figure 4:**
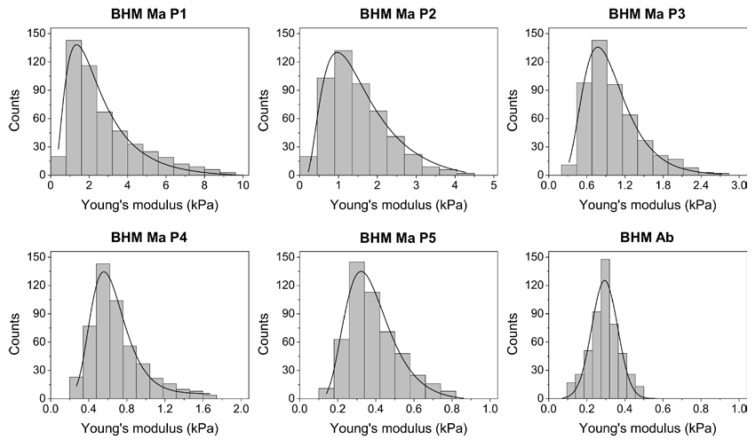
Histograms of the Young’s modulus values for the examined cells. Solid lines represent function fit to the data. In the case of BHM Ma cells, log-normal function was fitted, whereas for BHM Ab cells the Gaussian function was fitted. Note that the Young’s modulus data for BHM Ma cells becomes narrower and shifts towards lower values after each consecutive passage.

Finally, to examine the effect of endogenous pigment on the nanomechanical properties of the cells, atomic force spectroscopy (AFS) was employed. Figure 4 shows histograms of the Young’s modulus values obtained with AFS, whereas average values of the Young’s modulus determined for each cell sample are given in Table 1. 

As evident from the data, pigmented BHM Ma cells from first passage had the highest average value of the Young’s modulus, which was nearly ten times greater than that of non-pigmented BHM Ab cells, which had the lowest average value of the Young’s modulus. Noticeably, the average values of the Young’s modulus for BHM Ma cells became lower after each consecutive passage. This is indicated by shifting of the Young’s modulus values near the lower end of the histogram. Moreover, the distribution of the obtained Young’s modulus values for BHM Ma cells became more normal after each consecutive passage. In the case of cells from first passage, one could argue that these cells should have the lowest value of the Young’s modulus, since they were less attached and spread, and had a less developed actin cytoskeleton. However, these cells contained the largest amount of stiff melanin granules, which significantly increased the values of the Young’s modulus of these cells. Dependence graph between the Young’s modulus values and melanin content (Supplementary Materials Figure S1) indicates a clear correlation between pigmentation and elasticity of BHM cells. 

### 2.5. Force Mapping

**Figure 5:**
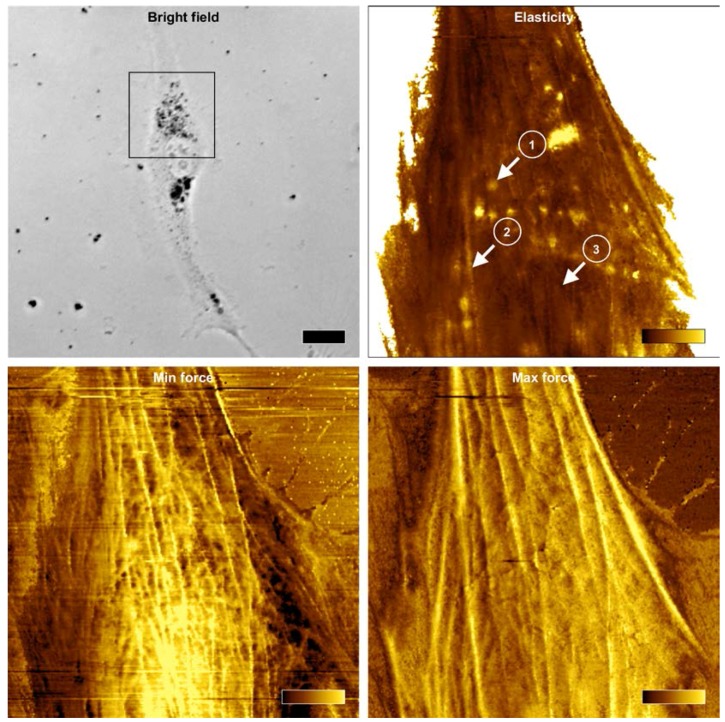
Force maps reconstructed from force curves obtained during PeakForce imaging. Bright field optical microscopy image (**upper left**) of a pigmented BHM cell taken during atomic force microscopy (AFM) analysis. The black frame in the image indicates scan area of 40 × 40 µm^2^ covered by AFM. Dark spots in the image are melanin granules. The scale bar for this image represents 20 µm. Elasticity map of the Young’s modulus values (**upper right**) of the cell with three different positions (marked with arrows) from which representative force curves were pulled and are shown in Figure 6. These positions refer to: melanin granule (1), actin bundle (2) and the nucleus (3). The color bar in elasticity map represents values of the Young’s modulus ranging from 0 to 10 kPa (dark-to-bright). Force error maps obtained for two different levels of force: minimum force (**lower left**) and maximum force (**lower right**). Minimum force corresponds to the value of force at the point of contact of the AFM probe with the cell, whereas maximum force corresponds to the maximum force obtained by the probe during indentation (graphically illustrated in Figure 6). Color bars in force error maps represent values of forces ranging from −110 to 50 pN for min force and from 0.4 to 0.6 nN for max force. Note that in the minimum force image, thin filaments are seen in the cortex of a cell, whereas in the maximum force image, thick actin bundles are visible, which are located deeper in the cell.

In addition to traditional force spectroscopy measurements, force mapping was performed employing atomic force microscopy (AFM). Representative elasticity map of a BHM Ma cell (Figure 5 upper right) clearly shows that high values of the Young’s modulus reported for pigmented melanoma cells were caused by stiff melanin granules in the cells. 

**Figure 6:**
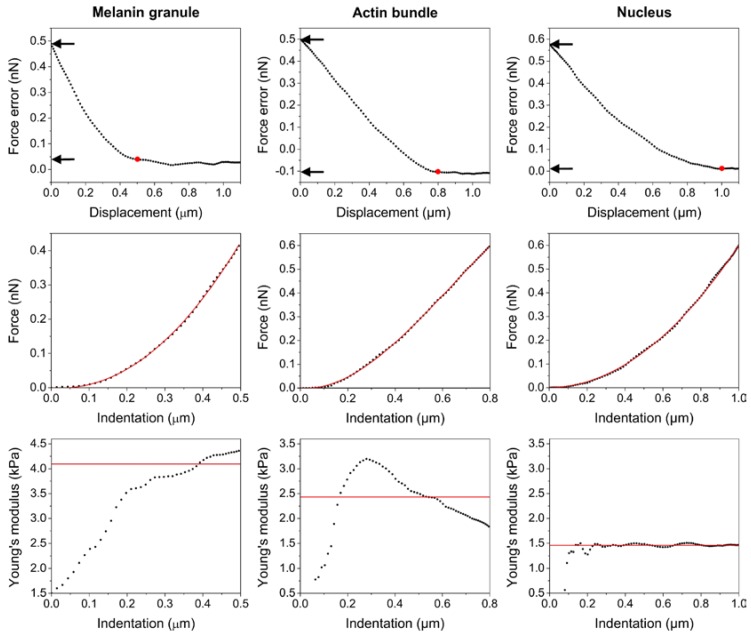
Representative force curves pulled from elasticity map for three different locations on the cell. Force error–displacement curves (**upper row**), followed by force–indentation curves (**middle row**) and Young’s modulus–indention curves (**bottom row**). Red dots in force error–displacement curves represent the point of contact of the AFM tip with the cell. Arrows point to the values of force on the y axis, which correspond to both minimum force (contact point) and maximum force (last point on the curve). Red lines in force–indentation curves represent fit of the theoretical model to the data points. Solid lines in pointwise modulus data represent the average values of the Young’s modulus calculated for each position. Note that the indentation depth differs significantly between the positions. It is lowest for a melanin granule and highest for the nucleus.

Example force curves pulled from elasticity map for three different locations on the cell (Figure 6) also showed that the pointwise modulus data differed significantly between the positions. For a location on top of a melanin granule, the values of the Young’s modulus were highest and increased with indentation depth. This indicates that during indentation, the AFM probe could not deform stiff melanin granule inside the cell and as a result the melanosome was pushed into deeper parts of the cell. On the other hand, pointwise modulus taken from a position on top of an actin bundle showed that the Young’s modulus values increased to a certain point and then decreased. This was most likely due to the fact that the actin bundle could resist the growing pressure caused by the indenting AFM probe to only a certain extent after which individual filaments started to disengage from the bundle. Finally, pointwise modulus taken from above the nucleus showed that the values of the Young’s modulus were lowest for this position and did not change over the entire indentation depth. 

These results indicate that the nuclear region of a pigmented BHM cell was mechanically very homogenous, whereas the remaining parts of the cell, in particular those containing melanin granules were very heterogeneous in mechanical means. Importantly, it is believed that the nuclear region is the most critical area of a cancer cell during transmigration through different barriers, since it has to undergo largest deformation proportionally to the cell body [20]. On the other hand, the level of actin cytoskeleton organization is viewed as the main contributor to the overall cell stiffness [21]. This is why cancer cells are in general softer than cells they originate from, hence during cancer transformation they undergo significant reorganization of their cytoskeleton, in particular actin cytoskeleton [22]. Force error images obtained for two different levels of force showed different cytoskeleton features formed by actin near the surface of the cell when compared to those inside the cell. Minimum force error image, corresponding to the cell surface (Figure 5 (lower left)) indicated thin filaments in the cortex of the cell, whereas maximum force error image, corresponding to the deeper parts of the cell (Figure 5 (lower right)) showed thick actin bundles in the cell. These images suggest that thick actin bundles deep in the cell should have the largest impact on the elasticity of BHM cells. Time lapse visualization of the pointwise modulus data (Supplementary Materials Movie S1) indicates that the contribution of actin cytoskeleton to the mechanical properties of a pigmented BHM cell is negligible. Only at low indentations actin filaments have a noticeable impact on cell elasticity, whereas at higher indentations, melanin granules dominate any influence of the cytoskeleton on the nanomechanical properties of a BHM cell.

## 3. Discussion

In this work, we have demonstrated that the nanomechanical phenotype of melanoma cells depends solely on the amount of endogenous pigment in the cells. Our findings indicate that melanin pigmentation should be carefully monitored when examining elasticity of melanoma cells in both in vitro and ex vivo studies. Importantly, nanomechanical properties of different cancer cells, including melanoma cells, have been extensively studied over the last years for the postulated role of cell elasticity in the process of metastasis [23]. It was even postulated that cell elasticity could be used as a diagnostic marker in the case of cancers in which traditional examination gives poor results [24]. Indeed, if applicable, this would be particularly helpful in the case of melanoma for which early diagnosis is of critical importance [25]. However, recent studies on melanoma nanomechanics yielded contradictory results, putting into question applicability of elasticity measurements in melanoma diagnosis. In one study, authors reported that two closely related human melanoma cell lines with different metastatic potential (WM115 and WM266-4) differed in their stiffness with the more aggressive, designated as WM266-4, being slightly softer. The authors attributed these differences to the highly flexible ridges found on the surface of metastatic melanoma cells [26]. In another study, researchers showed that metastatic melanoma WM239A cells were actually stiffer than non-metastatic WM115 cells [27]. In the analysis, cells were maintained on substrates with varying adhesion to reflect the morphological changes during different growth phases of melanoma. Interestingly, the biggest difference between metastatic and non-metastatic melanoma cells was observed for cells cultured on fibronectin, which promoted the cells adhesion and therefore their spread. On the other hand, when cultured on non-adherent substrates or small adhesive spots that limited cell spreading, the cells appeared to be much softer and the stiffness difference between metastatic and non-metastatic melanoma cells diminished. Although these studies delivered valuable data on cancer cell nanomechanics, they are of limited relevance to melanoma research. In both citied works, researchers did not analyze cells containing melanin pigment—a key feature of melanoma [28]. As demonstrated in our previous studies, induction of melanin pigmentation in melanoma cells in vitro dramatically modified nanomechanical properties of the cells [14,15]. Moreover, in the present work based on the analysis of melanoma cells ex vivo, we showed that the magnitude of the mechanical effect of endogenous pigment on the overall elasticity of the cells dominated any influence of actin cytoskeleton organization and level of cell spread. 

There is no doubt that cell cytoskeleton is the main contributor to cellular mechanics of normal and cancer cells [29]. However, as demonstrated by us, in the case of melanoma cells, melanin presence is the dominating factor responsible for the overall mechanical properties of the cells. It is important to stress that melanin synthesis is a heterogeneous process, which leads to different levels of cell pigmentation in both in vitro and in vivo [30,31]. For years the role of melanin in melanoma was under extensive scrutiny with the results so far being inconclusive. It was shown that melanin pigmentation can affect the outcomes of photodynamic therapy and radiotherapy of melanoma [32,33,34]. However, it remains unclear whether melanin presence has any impact on the invasive abilities of melanoma cells during metastasis. Intriguingly, most recent clinical studies point to possible relationship between pigmentation of melanoma cells and their aggressiveness [35,36]. In these studies, researchers found that amelanotic melanoma was associated with poorer patient survival than pigmented melanoma. However, possible mechanism that would be responsible for such a behavior of melanoma cells remains unknown.

In our recent study, we demonstrated that melanoma cells containing melanin pigment were less capable to penetrate a mechanical barrier in vitro than cells without melanin [37]. Detailed analysis revealed that melanin presence reduced deformation capabilities of the cells critical when passing through a narrow opening similar to that of an invaded basement membrane or an endothelial barrier. Importantly, melanin presence had no effect on key functions of melanoma cells making the effect exclusively mechanical in nature. Based on these observations, we postulated that melanin presence should limit the metastatic abilities of melanoma cells in vivo. Of course, other key cell parameters, such as the effect of cancer cells on their microenvironment [38], connexin-formed gap junctions [39] and cell migration [40] are important in the process of metastasis. However, takin into consideration the magnitude of the mechanical effect of melanin presence on the elasticity of melanoma cells, we strongly believe that cell elasticity may be the most critical parameter responsible for melanoma invasiveness. 

A recent paper published by Pinner and others may support our view that melanin presence reduces the metastatic capabilities of melanoma cells. Using intravital imaging, researchers found that pigmented melanoma cells were less likely to spread in mice than cells without melanin [41]. Moreover, the authors showed that melanoma cells could switch between pigmented and non-pigmented states, which consequently affected their metastatic behavior. The observed effect was attributed to the level of cell differentiation, hence in melanocytes—cells from which melanoma originates—melanin pigmentation may indicate the differentiation state of a cell. Therefore, according to the authors’ interpretation, a melanoma cell either migrates (when less differentiated) or synthesizes melanin (when more differentiated). Noticeably, the mechanical effect of melanin presence on the metastatic behavior of the cells was not taken into consideration at all. It should be emphasized, that unlike in melanocytes, in which melanin synthesis is regulated by different factors [42], and plays a specific biological role [43], melanin pigmentation in melanoma cells is highly deregulated [44]. Moreover, until now no study has demonstrated any important function of melanin in melanoma, whatsoever. 

## 4. Materials and Methods 

### 4.1. Cell Isolation and Culture 

Bomirski hamster melanoma (BHM) cells were derived by Bomirski from a spontaneous melanoma in Syrian hamster. Both cell types used in the analysis (BHM Ma and BHM Ab) originated from the same tumor and were described in detail by Bomirski and others in the following work [45]. Unlike BHM Ma cells, which become heavily pigmented after in vivo transplantation, BHM Ab cells are amelanotic. In the experiments presented here, small tumor scraps containing BHM Ma cells were transplanted in vivo by a subcutaneous injection as described previously [46]. After reaching a specific diameter [47] tumors were surgically excised and dissected into small (1–2 mm) fragments. 10–15 pieces were placed in tissue culture flasks and maintained in RPMI-1640 culture medium, supplemented with 10% FBS, containing antibiotics and incubated in a 5% CO_2_ humidified atmosphere at 37 °C. After 3–5 days tumor scraps were removed, melanoma cells were passaged and used in the analysis. Cells from each subsequent passage, which was performed every 3 days, were analyzed. Cells were maintain in culture until they did not contain any visible pigment. Non-pigmented BHM Ab cells were cultivated under similar conditions.

### 4.2. Electron Paramagnetic Resonance 

The enhanced specificity of melanin determination with EPR was obtained employing the so called ‘zinc effect’ [48]. For the analysis, cells were detached from culture dishes, pelleted, counted, incubated in high concentration of zinc acetate, frozen, and stored at 77 K. The number of cells for each sample was approximately 10^6^ cells and the final concentration of zinc ions was 50 mM. EPR examination was performed in liquid nitrogen, using a finger-type quartz dewar and EMX-AA spectrometer (Bruker BioSpin GmbH, Rheinstetten, Germany) operating at X-band with 100 kHz magnetic modulation. As a standard for melanin quantification, synthetic cysteine-dopa melanin at a concentration of 2.05 mg/mL was used. Detailed description of EPR analysis used in this work can be found elsewhere [49]. 

### 4.3. Proliferation Assay

For proliferation analysis cells from each passage were seeded into 24 well plates (one plate per passage and four wells per one time interval) and maintained in culture for six days. Every 24 h cells were trypsinized and counted. The assay was repeated three times for statistical analysis.

### 4.4. Atomic Force Microscopy 

AFM analysis of the cells was conducted using a Bruker BioScope Catalyst (Bruker Nano Surfaces, Santa Barbara, CA, USA) coupled with an inverted optical microscope (Axio Observer Z1 from Zeiss, Oberkochen, Germany). Measurements were performed on cells maintained in culture medium at 37 °C. Mechanical analysis of the cells was made in force spectroscopy mode. Before each cell was analyzed, the AFM probe was aligned at the cell center using bright field optical microscopy image at ×400 magnification. Once aligned, force curves from a grid of 5 × 5 points were collected at a rate of 1 Hz. 20 cells for each cell sample were analyzed. Force maps of the cells were obtained using the PeakForce Tapping mode with the PeakForce Capture turned on. This resulted in acquisition of a force curve in each pixel of an AFM image. For mechanical measurements soft cantilevers were used with a nominal tip radius of 20 nm and spring constant of 0.01 N/m, whereas for PeakForce imaging a relatively soft cantilevers with a nominal tip radius of 20 nm and spring constant of 0.68 N/m were chosen. For precise mechanical characterization spring constants of the used cantilevers were routinely determined based on the thermal tune procedure [50]. Analysis of force curves and reconstruction of force maps from the curves was made using AtomicJ software [51]. In brief, the collected force–displacement curves were first converted into force–indentation curves and fitted with the Sneddon model. In addition, each force curve was analyzed using the pointwise modulus approach, which is based on calculating the value of the Young’s modulus for each point of a force curve independently [52]. To avoid any substrate-induced effects, the correction for thin samples was made [53]. Detailed description of the mechanical analysis used in this work can be found elsewhere [54].

### 4.5. Confocal Microscopy

Analysis of the cytoskeleton was made on cells fixed with 3.7% formaldehyde, permeabilised with 0.1% Triton X-100 and blocked with 1% bovine serum albumin at room temperature. Cells were immunostained with mouse monoclonal anti-human α-tubulin IgG (Sigma-Aldrich, St. Louis, MO, USA) and Alexa Fluor 488-conjugated goat anti-mouse IgG (A110011, Life Technologies, Hong Kong, China), and counterstained with Alexa Fluor 568-phalloidin (Life Technologies, Hong Kong, China) and Hoechst 33342 dye for DNA stain (Sigma-Aldrich, St. Louis, MO, USA). Images were obtain using scanning laser confocal microscope (LSM 880 from Zeiss).

### 4.6. Statistical Analysis

Statistical significance of differences between mean values was assessed using the two-sample independent Student’s *t*-test at 95% confidence level. Statistical analysis was made using Mathematica 8.0 software (Wolfram, Oxfordshire, UK). 

## 5. Conclusions

Results obtained in this work demonstrate that neither organization of actin cytoskeleton nor the level of cell spread has a significant impact on the overall mechanical properties of melanoma cells containing endogenous pigment. Presence of stiff and hardly deformable melanin granules in melanoma cells dominates any influence of both cytoskeleton and level of cell spread. These findings together with the existing knowledge on cancer metastasis and cell nanomechanics may indicate an important role of melanin pigmentation in the process of metastasis of melanoma. Taking into consideration the magnitude of the mechanical effect of endogenous pigment on melanoma cell elasticity, we strongly believe that the nanomechanical phenotype of melanoma cells may be a reliable indicator of the cells’ metastatic behavior. 

## Figures and Tables

**Table 1:** Numerical values of the obtained results for BHM cells.

Cell Sample	Melanin Content (ng/Cell)	Doubling Time (h)	Young’s Modulus (kPa)
BHM Ma P1	0.24 ± 0.02	126.8 ± 20.3	2.27 ± 0.19
BHM Ma P2	0.13 ± 0.01	76.2 ± 15.2	1.46 ± 0.13 *
BHM Ma P3	0.081 ± 0.007	63.3 ± 11.8	0.89 ± 0.09 **
BHM Ma P4	0.038 ± 0.005	57.9 ± 7.1	0.63 ± 0.07 ***
BHM Ma P5	0.019 ± 0.003	43.9 ± 3.7	0.39 ± 0.05 ****
BHM Ab	-	22.1 ± 1.2	0.28 ± 0.01 *****

* Statistically significant vs. BHM Ma P1; ** statistically significant vs. BHM Ma P2; *** statistically significant vs. BHM Ma P3; **** statistically significant vs. BHM Ma P4; ***** statistically significant vs. BHM Ma P5. For all values *p* < 0.0001.

## Supplementary Materials

Supplementary materials can be found at http://www.mdpi.com/1422-0067/19/2/607/s1.

## Abbreviations

AFM/S	Atomic force microscope/spectroscopy
BHM Ma/Ab	Bomirski hamster melanoma Melanotic/Amelanotic
EPR	Electron paramagnetic resonance
LSCM	Laser scanning confocal microscope
