# Supplementary material for: Nanomechanical Phenotype of Melanoma Cells Depends Solely on the Amount of Endogenous Pigment in the Cells
Source: Int J Mol Sci. 2018 Feb 18;19(2):607. doi: 10.3390/ijms19020607 (PMC5855829; doi:10.3390/ijms19020607)
Supplement: Supplementary file 1 [file ijms-19-00607-s001.pdf]

Supplementary materials

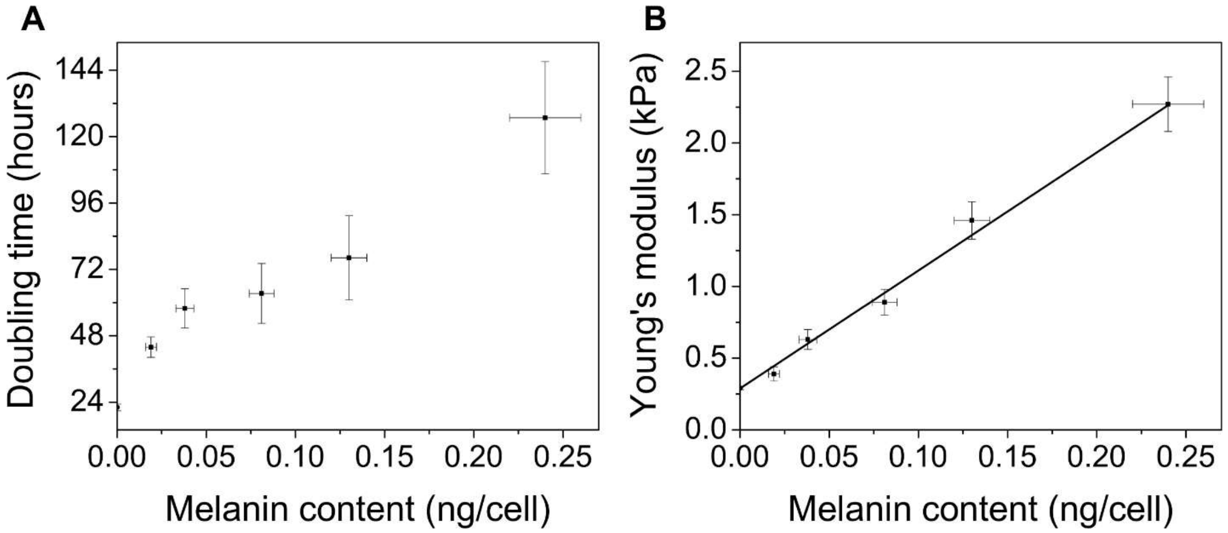

Figure S1. Relationship graphs between melanin pigmentation and cell proliferation as well as their elasticity. Melanin content vs. doubling time (**A**) followed by melanin content vs. Young's modulus values (**B**). Data points indicated with (■) symbol represent BHM Ma cells, whereas data points for BHM Ab cells are shown with (●) symbol. Note that the Young's modulus values depend linearly on the amount of melanin inside cells, whereas the relationship between the doubling time of the cells and their pigmentation is not linear.

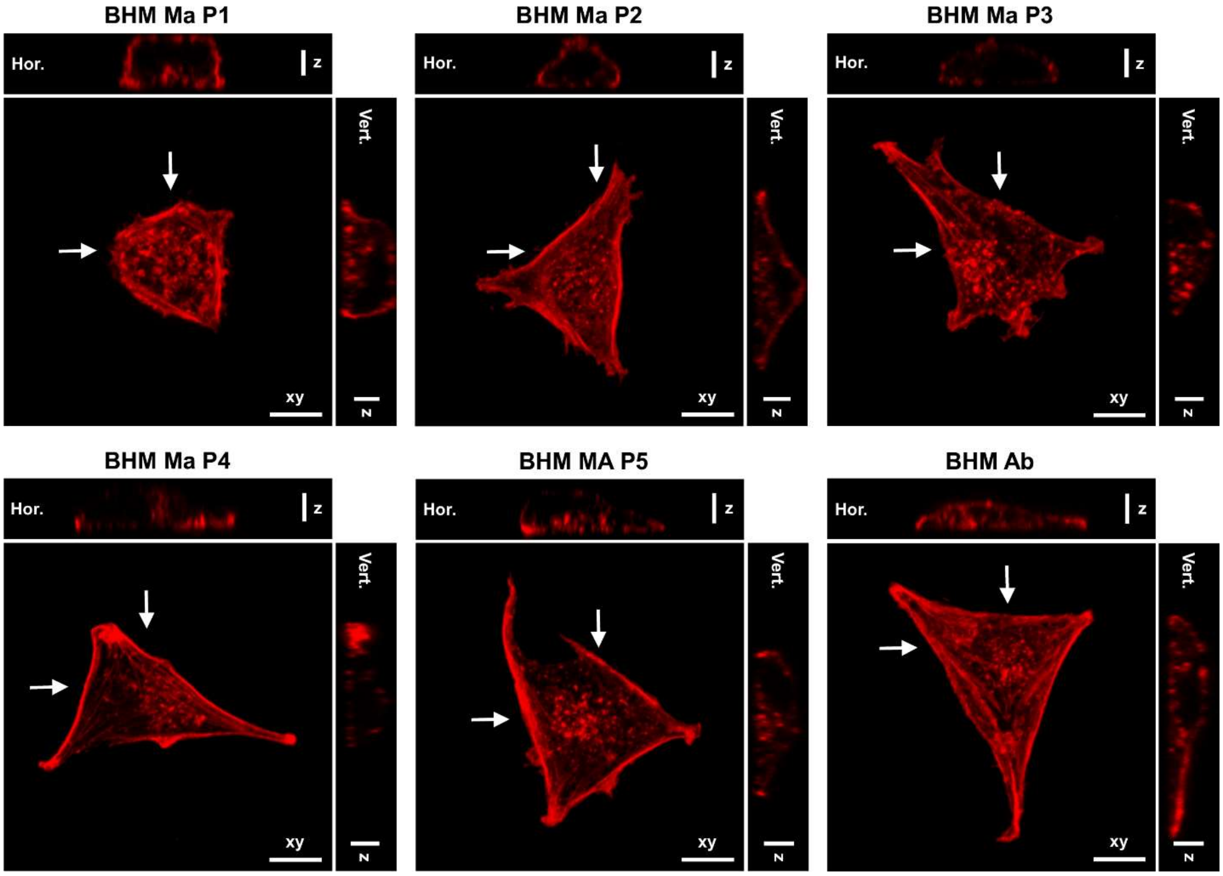

Figure S2. Cross sections of the cells made based on confocal microscopy analysis. Maximum intensity projection images of actin cytoskeleton (large images) and their cross sections (small images). Arrows show the direction of cross sectioning: horizontal arrows indicate sectioning in the horizontal direction and the corresponding cross sections are shown above the images, whereas vertical arrows indicate sectioning in the vertical direction and the corresponding cross sections are shown next to the images. Cross sections were made at the highest point of the cells. Scale bars for ‘xy’ indicate 10 µm, whereas for ‘z’ 5 µm. Note that BHM Ma cell from first passage was highest and less spread than cells from later passages and BHM Ab cell, which was lowest and most spread.

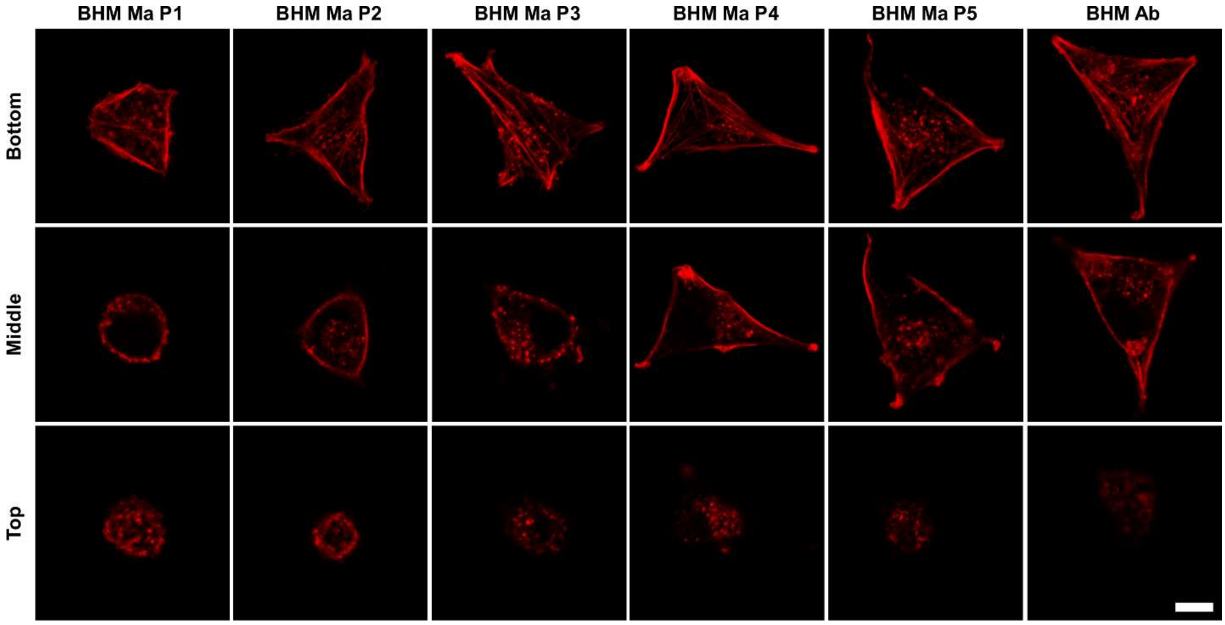

Figure S3. Organization of actin cytoskeleton in BHM cells. Confocal microscopy images of actin cytoskeleton taken at three different focusing levels: at the bottom of the cells near the glass coverslip (first row), in the middle of the cells (second row) and near the cells surface (third row). These images indicate different cytoskeleton features formed by actin inside the cells. At the bottom of the cells actin forms thick bundles, which are apparent in BHM Ma cells from all passages, however the bundle thickness of BHM Ma cells, starting from third passage is similar to that of a BHM Ab cell. In the middle of the cells actin forms filaments mostly near the cell edges, which are apparent in the case of BHM Ma cells starting from fourth passage. In the upper parts of the cells near the cell surface most actin was found for a BHM Ma cell from first passage, whereas BHM Ma cell from consecutive passages had fewer actin with BHM Ab cell having least actin near the cell surface. Note that BHM Ma cell from first passage exhibit the least developed cytoskeleton i.e. actin in this cell was mostly located near the cell membrane, whereas in cells from later passages and in a BHM Ab cell actin forms many filamentous structures both at the bottom and in the middle of the cells. Scale bar for all images indicate 10 µm.

Movie S1. Time-lapse presentation of the pointwise modulus data for a pigmented BHM cell. Each frame in the movie corresponds to the value of indentation, which is indicated by the counter at the top of the frame, whereas color bar represents values of the Young's modulus ranging from 0 to 20 kPa. Note that cytoskeleton features are noticeable in first few frames, which correspond to low indentation. At higher indentation, melanin granules dominate any influence of cell cytoskeleton to the elasticity of the cell.
